# Cord blood calcium, phosphate, magnesium, and alkaline phosphatase gestational age-specific reference intervals for preterm infants

**DOI:** 10.1186/1471-2431-11-76

**Published:** 2011-08-31

**Authors:** Tanis R Fenton, Andrew W Lyon, M Sarah Rose

**Affiliations:** 1 Department of Community Health Sciences, University of Calgary, 3280 Hospital Drive NW, Calgary, Alberta T2N 4Z6, Canada; 2 Nutrition Services, Alberta Health Services, 1403 29 Street NW, Calgary, Alberta T2N 2T9, Canada; 3 Clinical Biochemistry, Calgary Laboratory Services, 9, 3535 Research Rd. NW, Calgary, Alberta, T2L 2K8, Canada; 4 Clinical Biochemistry, Department of Pathology and Laboratory Medicine, University of Calgary, 9, 3535 Research Rd. NW, Calgary, Alberta T2L 2K8, Canada; 5 Research Excellence Support Team, Alberta Health Services, Alberta Health, Services, 1403 29 Street NW, Calgary,ABT2N 2T9, Canada

**Keywords:** Infant, premature, Infant, very low birth weight, reference values, reference ranges

## Abstract

**Background:**

The objective was to determine the influence of gestational age, maternal, and neonatal variables on reference intervals for cord blood bone minerals (calcium, phosphate, magnesium) and related laboratory tests (alkaline phosphatase, and albumin-adjusted calcium), and to develop gestational age specific reference intervals based on infants without influential pathological conditions.

**Methods:**

Cross-sectional study. 702 babies were identified as candidates for this study in a regional referral neonatal unit. After exclusions (for anomalies, asphyxia, maternal magnesium sulfate administration, and death), relationships were examined between cord blood serum laboratory analytes (calcium, phosphate, magnesium, alkaline phosphatase, and albumin-adjusted calcium) with gestation age and also with maternal and neonatal variables using multiple linear regression. Infants with influential pathological conditions were omitted from the development of gestational age specific reference intervals for the following categories: 23-27, 28-31, 32-34, 35-36 and > 36 weeks.

**Results:**

Among the 506 preterm and 54 terms infants included in the sample. Phosphate, magnesium, and alkaline phosphatase in cord blood serum decreased with gestational age, calcium increased with gestational age. Those who were triplets, small for gestational age, and those whose mother had pregnancy-induced hypertension were influential for most of the analytes. The reference ranges for the preterm infants ≥ 36 weeks were: phosphate 1.5 to 2.6 mmol/L (4.5 to 8.0 mg/dL), calcium: 2.1 to 3.1 mmol/L (8.3 to 12.4 mg/dL); albumin-adjusted calcium: 2.3 to 3.2 mmol/L (9.1 to 12.9 mg/dL); magnesium 0.6 to 1.0 mmol/L (1.4 to 2.3 mg/dL), and alkaline phosphatase 60 to 301 units/L.

**Conclusions:**

These data suggest that gestational age, as well as potentially pathogenic maternal and neonatal variables should be considered in the development of reference intervals for preterm infants.

## Background

In addition to the structural roles calcium, phosphate, and magnesium have in bone, these minerals also have key roles in most cellular, physiological and biochemical processes. Pediatric Societies recommend that preterm infants grow similar to the fetus [1-3], and maintain fetal concentrations in blood and tissues [1,2]. Experts on bone mineralization of preterm infants recommend that cord blood serum phosphate be maintained greater than 1.5 mmol/L (4.5 mg/dl) [4], 1.8 mmol/L (5.6 mg/dl) [5], or 1.9 mmol/L (5.8 mg/dL) [6], and alkaline phosphatase (ALP) be less than 400 [5] or 800 units/L [4]. To our knowledge only small studies have documented bone minerals related analytes (calcium, phosphate, magnesium, alkaline phosphatase, and albumin-adjusted calcium,) in cord blood serum of preterm infants [7-9] or fetal blood [10-13]. None of these studies have systematically evaluated the effect of potentially influential maternal and infant medical conditions on cord blood serum bone minerals (calcium, phosphate, magnesium) and related laboratory tests (ALP and albumin-adjusted calcium).

The purpose of our study was to 1) determine whether cord blood serum levels of bone minerals (cord blood serum calcium, phosphate, and magnesium), and related laboratory tests (ALP and albumin-adjusted calcium) vary with a) gestational age and/or b) maternal and/or neonatal variables, and to 2) estimate the cord blood serum reference intervals for healthy preterm infants born between 23 and 36 weeks of gestational age, that is, preterm infants who were not influenced by potentially pathogenic maternal or neonatal variables.

## Methods

This cross-sectional study was approved by the Conjoint Health Research Ethics Board, University of Calgary and Calgary Health Region, and conducted in Foothills Hospital, the regional high-risk maternal and neonatal referral centre for Southern Alberta between July 2003 and February 2005. Umbilical cord blood of the subjects was identified among samples routinely banked at delivery for all births (n = 7174). Candidate preterm infants (< 36-37 weeks) were identified from the admission lists to the neonatal intensive care unit. Candidate singleton term infants were randomly selected from the list of cord blood samples in July, September and January using random number lists. The serum was stored at -70 C prior to testing.

Informed consent was requested from the parents by telephone to analyze the blood sample and review the infant's chart for gestational age, and co-morbidities. Gestational age, measured in weeks completed, was based on primarily on maternal dates and early ultrasounds or the modified Ballard score [14] assessment. Size for gestational age was assigned using the 10^th ^and 90^th ^centiles using reference values [15].

702 infants, 634 preterm and 68 term infants were identified as candidates for this study. The parents of 28 infants refused inclusion of their babies (23 preterm (3.6%) and 5 term (7.6%)) in the study, and 63 parents were not able to be contacted in four attempts (54 preterm (8.3%) and 9 term (13.6%)). Infants were excluded from the study: 17 babies for asphyxia (2^nd ^Apgar ≥ 3 or cord pH < 7.0) [16], 13 (1.8%) for magnesium-sulfate treatment during labour [17], seven (1.0%) for anomalies, and 14 (2.0%) who died prior to hospital discharge.

Cord blood serum calcium, phosphate, magnesium and ALP (total ALP = from bone and liver) were determined on a Hitachi 917 (Roche Diagnostics Canada, Laval) using the manufacturers reagents and calibrators. Albumin-adjusted calcium was calculated using the bromocresol purple albumin binding reagent equation adjusted [calcium](mmol/L) = total [calcium](mmol/L) + 0.012 (39.9 - [albumin](g/L)[18]) since these values are useful to consider for subjects with hypoalbuminemia.

### Statistical Methods

The distributions of categorical variables observed were summarized as percentages by gestational age group (Table 1). Subsequently, for each analyte, a simple linear regression analysis was used to examine the relationship between the analyte (dependent variable) and gestational age in completed weeks (independent variable). In all regression models the intragroup correlation induced by twins and triplets was accounted for using a robust variance estimator for cluster correlated data [19], implemented using the cluster option in the regress command in Stata 11.

**Table 1:** Subject characteristics

Gestational Age (weeks)	23-27		28-31		32-34		35-36		All preterrm		Term	
	n	%	n	%	n	%	n	%	n	%	n	%
n	51	9%	133	24%	199	36%	123	22%	506	90%	54	10%
												
Male	26	51%	83	62%	103	52%	74	60%	286	57%	34	63%
Female	25	49%	50	38%	96	48%	49	40%	220	43%	20	37%
												
Diabetes												
None	50	98%	113	85%	187	94%	114	93%	464	92%	52	96%
Pre-existing	1	2%	8	6%	6	3%	3	2%	18	4%	1	2%
Gestational	0	0%	12	9%	6	3%	6	5%	24	5%	1	2%
												
Multiple Births												
Single	39	76%	81	61%	115	58%	76	62%	311	61%	54	100%
Twin	12	24%	41	31%	77	39%	43	35%	173	34%	0	0%
Triplet	0	0%	11	8%	7	4%	4	3%	22	4%	0	0%
												
Winter	27	53%	59	44%	114	57%	62	50%	262	52%	22	41%
												
PIH	1	2%	22	17%	31	16%	14	11%	68	13%	1	2%
												
SGA	49	96%	130	98%	192	96%	108	88%	27	95%	54	100%
												
Beta-methasone	41	80%	114	86%	113	57%	19	15%	287	57%	0	0%
												
Delivery												
Vaginal	24	47%	51	38%	97	49%	52	42%	224	44%	38	72%
Caesarean	27	53%	82	62%	101	51%	71	58%	281	56%	15	28%

A quadratic variable for gestational age was fitted to examine whether there was evidence of a non linear component. Subsequently, each of the potentially influential variables (maternal diabetes (none, pre-existing or gestational), multiple births (twin and/or triplet), pregnancy-induced hypertension (PIH), beta-methasone use, small for gestational age (SGA), gender, birth season (summer versus winter as a surrogate measure of vitamin D status) [20], delivery (caesarean vs. vaginal), and arterial pH) were added to gestational age one at a time in a multiple linear regression model (adjusting for correlated data as above) to assess for confounding and to determine whether these variables were statistically significant predictors of the analyte. Each variable was considered a confounding variable of the relationship between gestational age and the analyte if it changed the estimate of the slope between the analysts and gestational age by more than 10% when included in the model, or was considered a statistically significant predictor of the analyte after controlling for gestational age (if the p -value for the Wald statistic was < 0.05). Subsequently all potential confounding and significant variables were included in a multivariable model (i.e. multiple linear regression model) but removed if p > 0.05 or if no evidence of confounding by that variable. To assess the clinical relevance of a significant predictor variable in terms of the construction of the reference intervals, the magnitude of each coefficient was reported as a percent of the overall mean and standard deviation for that analyte.

There was apparent evidence of some confounding of the relationship between each analyte and gestational age by pH but this was found to be due to missing pH values (6%). Thus a variable was used to categorize the non-missing and missing blood pH into 3 categories (< or > 7.35 vs. missing), to allow examination of the effect of the missing data so that all the data could be used in the regression analysis.

For the generation of the reference intervals, when potentially pathogenic conditions were found to substantially affect the levels of the analyte, the individual babies with these conditions were omitted for that analyte. Potentially pathogenic conditions were defined *a priori *as the pathogenic variables that statistically significantly influenced the analyte, and whose influence, singly or combined, was greater than 10% of the mean or greater than 50% of the standard deviation. Reference intervals were calculated using the mean ± 1.96 standard deviations within strata defined by gestational age groups of 23-27, 28-31, 32-34, 35-36 and > 36 weeks.

## Results

The study sample included 506 preterm and 54 term infants. The majority of infants were 32 to 36 weeks of gestational age, but ranged from 23 to 42 weeks (Table 1). Most of the mothers and infants did not have any medical conditions, most were singleton births (65%), 4.8% were SGA, 52% were given at least one dose of beta-methasone, and 53% had caesarean births. The reference intervals for the various gestational age categories are shown in Table 2 (mmol/L) and Table 3 (mg/dl).

**Table 2:** Laboratory Reference Intervals by gestational age for phosphate, calcium, albumin-adjusted calcium, magnesium (mmol/L), and alkaline phosphatase (units/L)

Phosphate (mmol/L)					
*Gestational Age (weeks)*	*n*	*Mean*	*SD*	Lower limit*	Upper Limit*
23-27	51	2.1	0.3	1.6	2.6
28-31	119	2.0	0.3	1.4	2.7
32-34	191	2.0	0.2	1.5	2.5
35-36	119	2.0	0.3	1.4	2.6
> 36 weeks (term)	52	1.8	0.2	1.4	2.3
**Calcium (mmol/L)**					
23-27	51	2.5	0.2	2.0	3.0
28-31	115	2.6	0.3	2.0	3.1
32-34	176	2.6	0.2	2.2	3.1
35-36	110	2.6	0.3	2.1	3.1
> 36 weeks (term)	52	2.7	0.1	2.5	3.0
**Albumin-adjusted Calcium (mmol/L)**					
23-27	49	2.7	0.2	2.2	3.2
28-31	109	2.7	0.3	2.2	3.3
32-34	175	2.8	0.2	2.3	3.2
35-36	116	2.7	0.2	2.3	3.2
> 36 weeks (term)	51	2.8	0.1	2.6	3.0
**Magnesium (mmol/L)**					
23-27	48	0.79	0.07	0.65	0.94
28-31	127	0.79	0.10	0.58	0.99
32-34	193	0.76	0.08	0.60	0.92
35-36	120	0.76	0.09	0.58	0.93
> 36 weeks (term)	53	0.77	0.08	0.63	0.92
**Alkaline Phosphatase (units/L)**					
23-27	50	201	61	80	321
28-31	130	196	68	61	330
32-34	197	174	55	66	283
35-36	123	166	56	57	275
> 36 weeks (term)	52	159	49	62	256

* Lower limit is the mean - 1.96 SD and the upper limit is the mean + 1.96 SD

Infants were omitted from the development of the reference intervals when they had pathogenic conditions that were found to be influential to the results, based on *a priori *established criteria.

**Table 3:** Laboratory Reference Intervals by gestational age for phosphate, calcium, albumin-adjusted calcium, magnesium (mg/dL), and alkaline phosphatase (units/L)

Phosphate (mg/dL)					
*Gestational Age (weeks)*	*n*	*Mean*	*SD*	Lower limit*	Upper Limit*
23-27	51	6.5	0.9	4.9	8.2
28-31	119	6.3	1.0	4.3	8.2
32-34	191	6.1	0.8	4.6	7.8
35-36	119	6.1	1.0	4.2	8.0
> 36 weeks (term)	52	5.7	0.7	4.3	7.1
**Calcium (mg/dL)**					
23-27	51	10.0	1.0	8.1	11.9
28-31	115	10.2	1.2	8.0	12.5
32-34	176	10.5	1.0	8.6	12.4
35-36	110	10.4	1.1	8.3	12.5
> 36 weeks (term)	52	10.9	0.5	9.8	11.9
**Albumin-adjusted Calcium (mg/dL)**					
23-27	49	10.8	0.9	8.9	12.6
28-31	109	10.9	1.1	8.8	12.9
32-34	175	11.1	0.9	9.3	12.7
35-36	116	10.9	0.9	9.0	12.8
> 36 weeks (term)	51	11.2	0.5	10.2	12.2
**Magnesium (mg/dL)**					
23-27	48	1.9	0.2	1.6	2.3
28-31	127	1.9	0.3	1.4	2.4
32-34	193	1.8	0.2	1.5	2.2
35-36	120	1.8	0.2	1.4	2.3
> 36 weeks (term)	53	1.9	0.2	1.5	2.2
**Alkaline Phosphatase (units/L)**					
23-27	50	201	61	80	321
28-31	130	196	68	61	330
32-34	197	174	55	66	283
35-36	123	166	56	57	275
> 36 weeks (term)	52	159	49	62	256

* Lower limit is the mean - 1.96 SD and the upper limit is the mean + 1.96 SD

Infants were omitted from the development of the reference intervals when they had pathogenic conditions that were found to be influential to the results, based on *a priori *established criteria.

### Phosphate

Serum phosphate was negatively related to gestational age (Table 4). No variables confounded the relationship between cord blood serum phosphate and gestational age, but three variables were significant predictors of cord blood serum phosphate: triplets, SGA, and PIH. These three variables were entered into the multivariable regression model; triplets and PIH remained significant in the multivariable model (Table 4). The effect of both triplets and PIH decreased the cord blood serum phosphate level, and triplets surpassed the preset change relative to the standard deviation (Table 4), so the 22 triplets (3.9%) were omitted for the development of the reference intervals.

**Table 4:** Estimates from the multiple regression models and magnitude of influential variables in the multivariable models

*Analyte*	*Variable*	*Coefficient*	*Standard Error*	*p - value*	*% mean*	*% SD*
**Phosphate** **(mg/dl)**	Gestational Age (weeks)	-0.057	0.01	< 0.001		
	Twin	-0.10	0.09	0.28	-1.6	-11.3
	Triplet	-0.51	0.15	0.001	-8.3	-57.6
	PIH	-0.29	0.11	0.009	-4.7	-32.7
	Intercept	8.07	0.33	< 0.001		
						
**Calcium**	Gestational Age (weeks)	0.054	0.01	< 0.001		
**(mg/dl)**	Twin	-0.23	0.01	0.023	-2.2	-22.8
	Triplet	-0.40	0.17	0.017	-3.9	-39.6
	SGA	-0.33	0.13	0.012	-3.2	-32.7
	PIH	-0.38	0.11	0.001	-3.7	-37.6
	Intercept	8.76	0.36	< 0.001		
						
**Albumin**	Gestational Age (weeks)	0.023	0.01	0.029		
**Adjusted**	SGA	-0.30	0.11	0.009	-2.7	-32.4
**Calcium**	PIH	-0.38	0.10	< 0.001	-3.5	-41.1
**(mg/dl)**	Caesarian delivery	-0.28	0.09	0.001	-2.6	-30.3
	Intercept	10.41	0.36	< 0.001		
						
**Magnesium**	Gestational Age (weeks)	-0.007	0.002	0.006		
**(mg/dl)**	Twin	-0.069	0.025	0.006	-3.7	-31.9
	Triplet	0.023	0.042	0.58	1.2	10.6
	Intercept	2.1	0.08	< 0.001		
						
**Alkaline**	Gestational Age (weeks)	-3.69	0.74	< 0.001		
**Phosphatase**	Intercept	299	25	< 0.001		
**(units/L)**						

### Calcium

Serum calcium was positively related to gestational age (Table 4). On individual analysis for calcium, four variables were predictive of calcium in addition to gestational age: multiple births, delivery, SGA, and maternal PIH; no variables confounded the association with gestational age. The variables indicating multiple births and method of delivery were highly correlated since multiple births were more likely to be delivered by caesarean than singleton births, so these two variables could not be included in the multivariable model simultaneously. We chose to enter multiple births rather than delivery since it made more sense clinically, and the variables multiple births, SGA, and maternal PIH were statistically significant predictors in the multivariable model (Table 4) after adjusting for gestational age. Although these three variables were statistically significant predictors of decreased calcium levels, none of them individually surpassed indicators of influence unless infants had two of the three conditions: triplets, SGA, and being from a pregnancy with PIH (Table 4). Two of the three conditions occurred among only 48 babies (8.6%), and these infants were omitted for the development of the reference intervals.

### Albumin-adjusted Calcium

Total cord blood serum calcium was positively related to cord blood serum albumin, and albumin-adjusted cord blood serum calcium was related to gestational age (Table 4). On individual analysis for albumin-adjusted calcium with gestational age, four variables were predictive: multiple births, maternal PIH, SGA, and delivery; no variables confounded the association with gestational age. Multiple births were no longer predictive in the multivariable model (Table 4). The conditions of caesarean delivery, maternal PIH and SGA exceeded the preset criteria for influence when infants had at least two of the three conditions (Table 4), which occurred among 64 infants (11.4%), and they were omitted from the reference ranges.

### Magnesium

Serum magnesium was negatively related to gestational age (Table 4). Multiple birth (twins and triplets), and beta-methasone use were confounding factors for gestational age, and multiple births was also significant predictor of lower cord blood serum magnesium levels. However, beta-methasone use was no longer predictive upon multivariable analysis. Although there was a suggestion of a quadratic relationship between gestational age and cord blood serum magnesium this was not in evidence in the multivariable analysis. Although multiple birth infants had significantly lower cord blood magnesium, it did not surpass the preset change of the mean or standard deviation (Table 4), so no infants were omitted from the reference intervals.

### Alkaline phosphatise

ALP was negatively related to gestational age (Table 4). On individual analysis for ALP, no variables were predictive of ALP or confounded the association with gestational age.

Gender, maternal diabetes, and birth season were not significant predictors nor confounders for any of the analytes.

## Discussion

Serum phosphate, calcium, magnesium and ALP varied with gestational age, cord blood serum phosphate, magnesium and ALP negatively and calcium positively. The existence of these relationships between cord blood bone minerals levels (serum calcium, phosphate, and magnesium), and related laboratory tests (ALP, and albumin-adjusted calcium), with gestational age, and other neonatal variables (multiple birth, maternal PIH, and small size for gestational age) suggests that these factors should be considered for the development of preterm reference intervals.

Our ranges of preterm cord blood phosphate and calcium (Tables 2 and 3) are compatible with expert recommendations [5,6]. In contrast, our preterm cord blood range of ALP are considerably lower than expert recommendations to maintain preterm infant ALP less 400 [5] or 800 units/L [4]. The cord blood ALP reference range may not be a relevant goal for the care of the preterm infant since the postnatal levels are considerably different from the cord blood levels, and may not be achievable in clinical care of preterm infants.

These results come from a single center, which could be influenced by local events or lab methodology. Therefore these results should be verified and validated by larger multicentre studies.

Albumin-adjusted calcium values are useful to consider for subjects with hypoalbuminemia, since non-adjusted total calcium can be reduced by a lower albumin-bound fraction, while the ionized physiologically active portion may be normal. Therefore, when cord blood serum ionized calcium is not available, or not appropriate, the use of albumin-adjusted calcium is superior to the use of total calcium in hypoalbuminemic patients. Measurement of ionized calcium is altered by changes in cord blood serum pH [21], and therefore its utility in cord blood is hampered since pH can be variable in cord blood.

Although reference intervals are useful to consider when evaluating the health status of any group, they may or may not define ideal health status. Research is needed to determine whether these reference ranges define the ideal serum levels for preterm infants, or whether the expert recommendations for cord blood serum phosphate (maintained greater than 1.8 mmol/L [5]), and/or ALP (less than 400 [5] units/L may be superior goals and/or more relevant. Reference intervals from cord blood at preterm births are generated from infants who are born prematurely, and for that reason may not be in a state of ideal health. However, the fetus is considered to be the reference source for the establishment of the goals for normal concentrations of nutrients in the blood and tissue [1,2] and cord blood provides a convenient opportunity to sample fetal blood.

Serum calcium is usually maintained within a limited range *in vivo *by homeostatic mechanisms. It is interesting that our preterm lower limits for calcium extended lower than the term lower limits, which could be related to the very rapid calcium deposition in bone mid-gestation. Preterm infants are prone to hypercalcaemia in the neonatal intensive care unit, which can be seen secondary to hypophosphatemia [22,23]. Our findings suggest that cord blood serum calcium up to 12.4 mg/dl (3.1 mmol/L) is acceptable given these reference intervals.

Serum calcium and phosphate were influenced by multiple births; phosphate was significantly lower for triplets while cord blood serum calcium was significantly lower for twins. These effects were likely due to limited placental supply due to limited nutrient supply to the multiple fetuses.

In comparison to previous reports, our values for cord blood serum phosphate were almost identical to four other studies [7,8,12,13], but were lower than the small study from Japan [9]. Our values for cord blood serum calcium were similar to another cord blood report [7], and slightly higher than five other studies [8,9,11-13]. Our magnesium values were slightly lower than one study [8], and higher than another [13]. Compared to our ALP values, fetal levels have been reported as similar [11] or much higher [10,12].

Some previous work regarding analyte relationships with gestational age agrees with our findings while some others do not. Moniz et al also noted an increase in cord blood serum calcium with increasing gestational age among the foetuses assessed using fetoscopy [11]. Seki et al [9] reported a more dramatic negative relationship between phosphate and gestational age. In terms of ALP, other researchers [10,12] also observed decreases in ALP with gestational age among the preterm fetuses.

Although most literature regarding phosphate for preterm infants emphasizes phosphate's role in bone mineralization, phosphate also has roles in glucose metabolism. Serum phosphate decreases in response to infused glucose in adults, [24], presumably due to it entering cells in response to insulin secretion. Further, low cord blood serum phosphate may limit clinical stability since it is required for glucose tolerance, tissue sensitivity to insulin [25], as well as glucose-induced insulin secretion [26].

## Conclusions

Cord blood bone mineral levels (cord blood serum calcium, phosphate, and magnesium) and related laboratory tests (albumin-adjusted calcium and alkaline phosphatase) were related to gestational age, the several of them (calcium and phosphate) were also related to pregnancy-induced hypertension, size for gestational age and multiple birth. Therefore, it seems desirable to consider these variables and how they co-vary with gestational age in the development of reference intervals. Further study is required to define the optimal cord blood serum levels of these analytes for preterm infants.

## Abbreviations

(ALP): Alkaline phosphatise; (PIH): pregnancy-induced hypertension; (SGA): small for gestational age.

## Competing interests

The authors declare that they have no competing interests.

## Authors' contributions

TRF and AWL designed the study; MSR designed and carried out the statistical analysis in consultation with TRF and AWL. TRF drafted the initial version of the manuscript with input from AWL and MSR. AWL and MSR read and approved the final manuscript.

## Pre-publication history

The pre-publication history for this paper can be accessed here:

http://www.biomedcentral.com/1471-2431/11/76/prepub
